# Bioinspired Temperature-Responsive Multilayer Films and Their Performance under Thermal Fatigue

**DOI:** 10.3390/biomimetics3030020

**Published:** 2018-08-01

**Authors:** Nikolaos Athanasopoulos, Nicolaos J. Siakavellas

**Affiliations:** Department of Mechanical Engineering and Aeronautics, University of Patras, 26500 Patras, Greece; siakavel@mech.upatras.gr

**Keywords:** responsive materials, smart materials, bioinspired materials, nonliving plant tissues, anisotropy, thermal fatigue, microstructure, 4D printing, additive manufacturing

## Abstract

The structure of certain nonliving tissues determines their self-shaping and self-folding capabilities in response to a stimulus. Predetermined movements are realized according to changes in the environmental conditions due to the generated stresses of the multilayer anisotropic structure. In this study, we present bioinspired responsive anisotropic multilayer films and their fabrication process which comprises low-cost techniques. The anisotropic multilayer materials are capable of deforming their geometry caused by small temperature changes (<40 °C). The mismatch in the thermo-mechanical properties between three or more anisotropic thin layers creates responsive materials that alter their shape owing to the developed internal stresses. The movements of the material can be controlled by forming anisotropic homogenous metallic strips over an anisotropic thermoplastic layer. As a result, responsive multilayer films made of common materials can be developed to passively react to a temperature stimulus. We demonstrate the ability of the anisotropic materials to transform their geometry and we present a promising fabrication process and the thermal fatigue resistance of the developed materials. The thermal fatigue performance is strongly related to the fabrication method and the thickness of the strips. We studied the thermal fatigue performance of the materials and how the thermal cycling affects their sensitivity, as well as their failure modes and crack formation.

## 1. Introduction

Advances in materials technology have the potential to greatly affect a plethora of applications in different sectors. Urgent needs to be fulfilled are the development of low-weight structures, the integration of different functionalities and sensing abilities, as well as the energy efficiency and financial feasibility in different applications.

**Figure 1:**
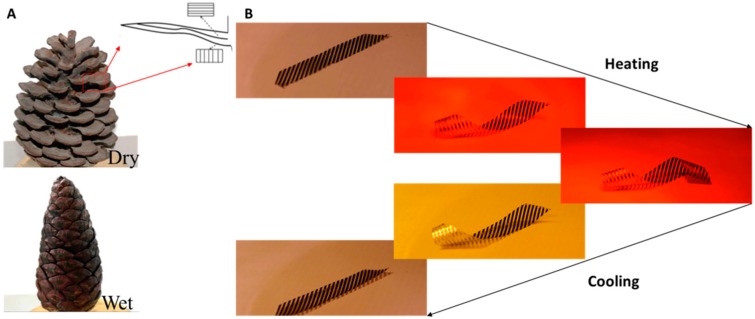
(**A**) Wet and dry pine cone, and its multilayer structure. (**B**) Response of the developed anisotropic film during the heating and cooling phase.

In nature, extremely complex movements can be realized through the materials’ self-shaping and self-folding capabilities in response to a stimulus [1,2,3,4,5,6,7,8,9]. The nonliving tissues of various plants are designed to undergo predetermined shape transformations through their anisotropic fibrous structure [1,2,3,6,7,8,9]. The coefficients of hygroscopic expansion are the corresponding parameters characterizing such changes in the physical dimensions of the plants’ nonliving tissues. Pine cones drastically transform their shape using only their anisotropic structure and the mismatch of the coefficients of hygroscopic expansion [1] (Figure 1A). This simple mechanism leads to the bending of the scales, which consequently opens the cone. This system can be regarded as a hygrosensitive bilayer material [1,3].

The mechanistic behavior/transformation of the aforementioned nonliving tissues inspired various researchers and can be imitated through the use of multilayered fibrous anisotropic materials, anisotropic nanocomposites, pre-stressed sheets, and nanoreinforced multilayer hydrogels [10,11,12,13,14,15,16,17,18]. The geometry of these materials can be transformed under humidity or temperature stimulus or both, whereas their initial and final shapes can be determined by the geometry, the homogeneous or nonhomogeneous nature of the materials’ structure, as well as the anisotropic nature of the different layers. Folding structures have been developed using shape-memory alloys (SMAs) in order to control their shape [18]. Apart from the well-known SMAs and shape-memory polymers (SMPs), three-dimensional (3D) printed hydrogel architectures have been developed. The shape shift of the biomimetic four-dimensional (4D) printed materials is actuated through the anisotropic swelling behavior in water. Various parameters control the shape transformation of the material, such as the filament size, orientation, and interfilament spacing [13]. Moreover, the swelling mechanism has been used to produce hygroscopic multilayer composites that control the orientation of microplatelets [14]. Other researchers use layer-by-layer (LBL) techniques for the fabrication of polymeric multilayers that are capable of driving shape transformations in response to environmental humidity and temperature variations. In this case, a hydrophilic multilayer is stacked with a less responsive carbon nanotube layer. The differential swelling of the two LBL films results in reversible out-of-plane deformations [11]. Moreover, complex flower structures have been developed from two-dimensional (2D) flat anisotropic polymeric sheets, whose shape-shifting behavior is enabled by the coefficient of thermal expansion (CTE) mismatch of the layers [19]. Other, techniques use 3D printed layers under tension and control their shape through the glass transition temperature of the materials and the applied stress [12]. Moreover, multilayer materials with anisotropic properties have been developed by the authors in order to actuate smart patterned surfaces to passively control the temperature of a body [16].

In this study, the deformation of the developed multilayer anisotropic materials is reversible and repeatable. The movements of these materials can be controlled by forming anisotropic homogenous metallic strips over an anisotropic thermoplastic layer. Owing to their large deformation, one of the most important parameters that affects their integrity is their performance under thermal fatigue conditions. The thermal fatigue performance is strongly related to the fabrication method. The proposed responsive multilayer anisotropic materials/films are able to passively react under temperature stimuli by transforming from 2D to 3D complex shapes. Here, we present a combination of low-cost fabrication processes for the development of anisotropic multilayer films in which a temperature change generates large deformations; this behavior is attributed to the mismatch in the CTE and to the anisotropic structure of the multilayer film, which causes the material transformation. Consequently, the thermal fatigue resistance of the developed materials regulates the potential of the material to be used in engineering applications. Three different anisotropic multilayer films with a different thickness and layer sequence were tested under thermal fatigue; we investigated the types of failure and crack formation using scanning electron microscopy (SEM). In addition, we present the fabrication process step by step, which yielded very good results regarding the thermal fatigue of the developed materials. A cold and hot rolling press, as well as etching techniques, have been employed in the fabrication process. These techniques are reliable and have been adopted for decades in the electronics industry. Furthermore, the layers of the multilayer responsive material consist of common and low-cost materials. For these reasons, we believe that the proposed fabrication methodology could be upscaled and be cost-effective.

## 2. Materials and Methods

### 2.1. Structure of the Material and Shape Transformation

The films consist of passive and responsive/active regions (black color) (Figure 1B). The sensitivity of the film is related to the density of the strips, the CTE of the different materials, and the degree of the orientation of the polyethylene (PE). The high CTE mismatch between the polymeric layers and the metallic strips creates materials that are very sensitive to temperature and alter their shape drastically owing to the developed internal stresses and the anisotropic nature of the material. The deformable regions are very responsive to temperature, presenting extremely large deformations. The spatial distribution of these strips and their direction determine the shape of the film as a function of temperature. Therefore, the areal density of the strips, the thickness, and the thermo-mechanical properties of the various materials determine the entire geometry transformation, thus enabling their a priori design.

The multilayer structure of the film differs for each region; however, the following layers must be combined to achieve proper function: one anisotropic low-CTE layer (strips), one anisotropic high-CTE layer (oriented PE), and one layer of adhesive. Moreover, we can incorporate more layers in order to achieve higher deformation, better thermal fatigue performance, and durability.

Figure 1B shows the response of a film that has been designed to be transformed to a helix. In this case, the principal axes of the oriented PE and those of the aluminum strips coincide in a 45° direction. The maximum temperature is ≈55 °C. Supplementary Videos S1, S2, and S3 show the transformation of the multilayer films with different anisotropic properties and geometries.

### 2.2. Fabrication Process

**Figure 2:**
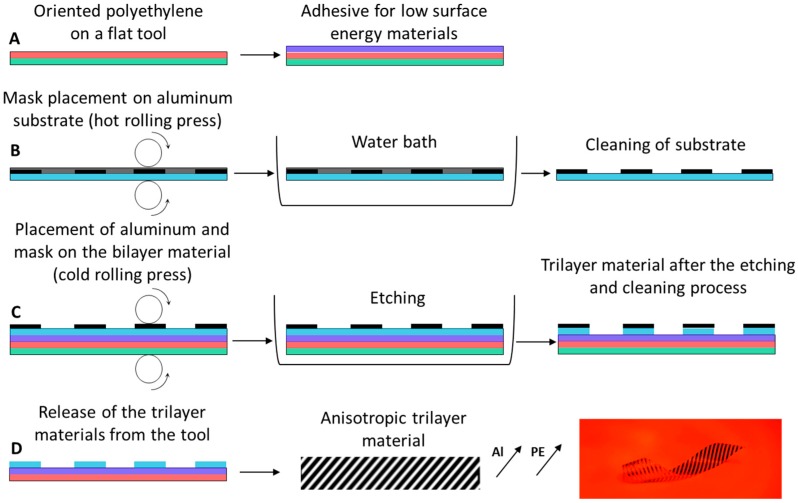
Fabrication process of the bioinspired films. (**A**) Fabrication of the bilayer material over a flat tool. (**B**) Mask placement over the aluminum film using a hot roll press and cleaning. (**C**) Cold roll pressing of the different layers and strip formation using etching techniques. (**D**) Cleaning and demolding. Al: Aluminum; PE: Polyethylene.

A two-component adhesive for low-surface energy plastics (methacrylate- and trimethylenediamine based resins (NEOTEX S.A., Greece) were used and applied on the oriented PE (Figure 2A). It should be mentioned that any adhesive for low-surface energy plastics can be used. The mask was attached over an aluminum film under pressure using a hot rolling press (PEAK PP330, PEAK, UK) at 180 °C in order to form the strips in the desired directions (Figure 2B).

The substrate of the mask was removed in a water bath and (Figure 2B) the masked aluminum film was pressed together with the bilayer material using a cold rolling press (Figure 2C). Then, the aluminum strips were formed by employing a chemical etching technique using a ferric chloride solution at 40 °C for <20 min. The fabricated multilayer materials were cleaned using water and acetone ≥99.5% (Figure 2C,D). The multilayer film was removed from the flat aluminum tool (Figure 2D). The fabrication process comprises low-cost manufacturing techniques, and different thermoplastic and metallic materials can be used.

### 2.3. Developed and Tested Anisotropic Multilayer Films

**Figure 3:**
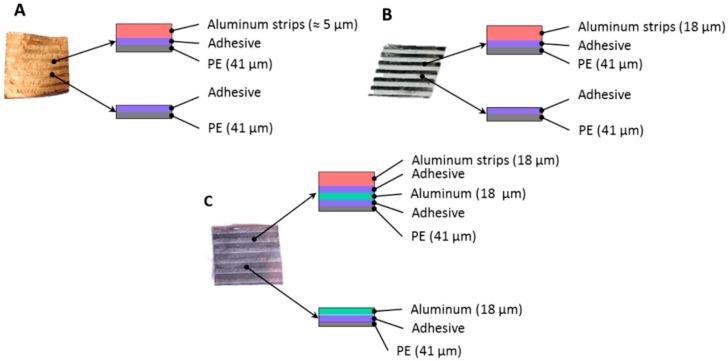
Test specimens (≈11 mm × 11 mm) for scanning electron microscopy (SEM) characterization. (**A**) Representative aluminum strips (5 μm) over a bilayer material. (**B**) Representative aluminum strips (18 μm) over a bilayer material. (**C**) Representative aluminum strips (18 μm) over a trilayer material. PE: Polyethylene.

The sequence of the anisotropic layers and the strips of the passive and responsive/active regions that were used are shown in Figure 3. Three different films were developed and tested. The first multilayer material consists of aluminum strips (thickness ≈ 5 μm) over a bilayer material (41 μm oriented PE and adhesive) (Figure 3A). The second multilayer film consists of aluminum strips (thickness = 18 μm) over the same bilayer material (Figure 3B). The third multilayer material consists of aluminium strips (thickness = 18 μm) over a trilayer material (41 μm PE, adhesive, 18 μm aluminum film) (Figure 3C).

The multilayer materials were tested under thermal cycling fatigue; we measured their deflection at the beginning of the test and after *N* thermal cycles. The failure modes were examined using a JEOL 6300 scanning electron microscope (JEOL, USA).

### 2.4. Thermal Cycling Measurement Setup

The scope of this experimental procedure is to evaluate the performance of the bioinspired film under accelerated thermal fatigue. An experimental setup was developed to study the degradation of the mechanical properties and the shape-shifting of the film. During the transformation of the material, any degradation in the polymeric layers would result in a different shape. Moreover, any failure of the layers or any interlaminar failure between the strips and the substrate (PE) would result in the shape-shifting of the film.

**Figure 4:**
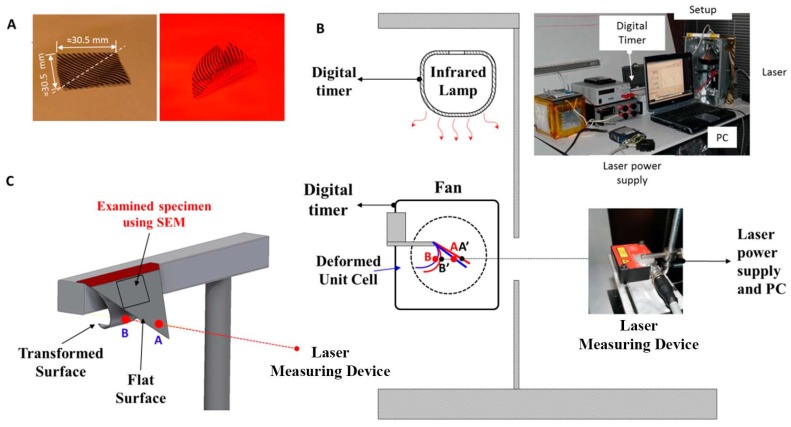
Dimensions of the tested multilayer film and experimental setup. (**A**) Representative anisotropic multilayer material at ≈20 °C and bending of the film under infrared heating at ≈55 °C. (**B**) Apparatus of the thermal fatigue test. (**C**) Three-dimensional drawing of the multilayer material and distance measurement between the points A and B. Points A’ and B’ represent the measured points after the thermal cycling test.

We fabricated three different multilayer materials with dimensions (30.5 mm × 30.5 mm) (Figure 4A). We cut the film in half (from one corner to the other). The first half was the healthy material and the other half was tested under thermal cycling. The multilayer material can be cut without damages using sharp scissors or a roller cutter. Each triangular specimen was placed between two plastic strips (sandwich structure).

The experimental setup (Figure 4B) consisted of an infrared (IR) lamp (Philips BR125 IR 250 W, E27, 230–250 V), a fan, a digital timer and counter, a power supply, a laser measuring device (Micro-Epsilon CS3, Micro-Epsilon, USA), a reflector curtain, and the tested anisotropic multilayer film (Figure 4A). The digital timer controlled the operation time of the IR lamp and that of the fan. The IR lamp heated the film until it reached its final transformation position. Immediately after the heating phase, the film was cooled using a fan as a cooler, and it returned to its initial position. A laser measuring device was continuously recording the distance between point A and point B (Figure 4B,C) during the cooling phase. A black mat spray was applied at the edge of the specimen (near point A) in order to record the measured distance as accurately as possible. The black spray forms a mat surface that minimizes the reflections of the laser beam. Any change in the recorded distance during cooling or any change in the measured distance of the initial position (A) or final position (B) would indicate that the mechanical properties of the multilayer material have been degraded or that a failure has occurred.

We cut smaller specimens (≈11 mm × 11 mm) and we scanned their entire surface using SEM of both the healthy and the fatigued multilayer materials, (detail of Figure 4C). The three pairs of specimens were examined through SEM.

## 3. Results and Discussion

We studied the response of different anisotropic multilayer films and how their geometry has been affected by the degradation of the mechanical properties due to the rapid thermal fatigue conditions. Through the experimental methodology, we intended to examine the performance of the films and to identify the failure modes of the material. Figure 4A shows the dimensions of the manufactured multilayer materials and the orientation of the aluminum strips. During the heating stage of the thermal fatigue test, the film was bended as shown in Figure 4A.

### 3.1. Thermal Cycling Fatigue

**Figure 5:**
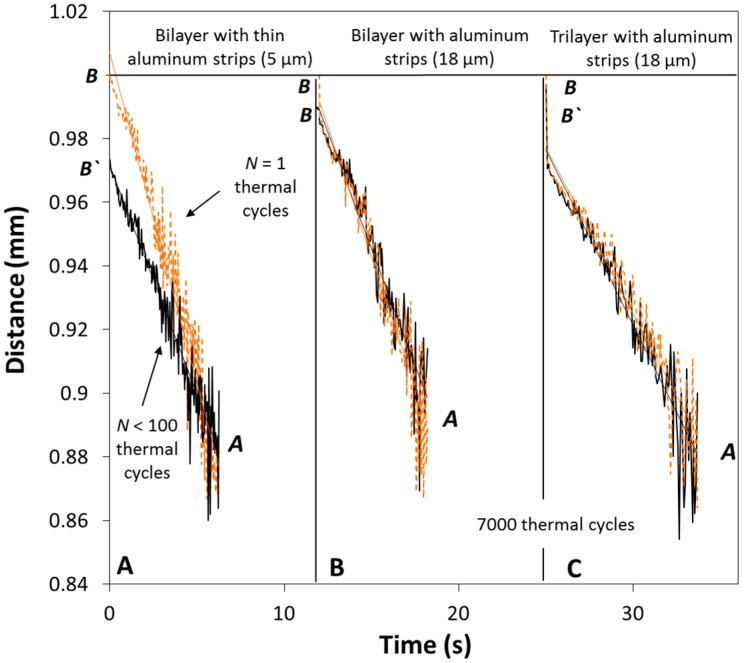
Distance measurements of the position of the tested multilayer film from point B to point A during the cooling stage after *N* thermal cycles under steady-state conditions. (**A**) Aluminum strips (5 μm) on a PE substrate. (**B**) Aluminum strips (18 μm) on a PE substrate. (**C**) Aluminum strips (18 μm) on a trilayer film with an aluminum substrate.

Figure 5 shows the measured distance versus time at the cooling phase after a certain number of thermal cycles for the three different developed films. The orange line represents the measured distance of the film during its first response (first thermal cycle), whereas the black line represents the recorded distance from point B to point A after a certain number of thermal cycles (*N*th thermal cycle). Any change in the final position of the measured point B to point B’ (Figure 4B) was indicative of the fact that the mechanical properties of the multilayer material had been degraded and that the shape of the film had changed.

#### 3.1.1. Aluminum Strips (5 μm) on a Polyethylene Substrate

The distance between the locations of the final position (Figure 5A) of the first specimen at the beginning (point B) and at the end (point B’) of the thermal cycling test for less than 100 thermal cycles changed by B_*Ν* = 1_ − B’_*Ν* = 7000_ = 2.8 mm. As a result, the inclination of the curve (from point B or B’ to point A) of the measured distance versus time at the beginning of the test and at 100 thermal cycles changes significantly (Figure 5A). Extensive cracks were observed and as a result, the response of the multilayer material decreased. The heating rate for the thinner specimen was 4 s and the cooling rate was 5.5 s.

#### 3.1.2. Aluminum Strips (18 μm) on a Polyethylene Substrate

The distance between the location of the final position (Figure 5B) of the second specimen during the beginning and the end of the test (after 7000 thermal cycles) changed by B_*Ν* = 1_ − B’_*Ν* = 7000_ = 1.3 mm. Moreover, we may observe that the inclination of the curve (from point B or B’ to point A) of the measured distance versus time at the beginning of the test (orange line) and after 7000 thermal cycles (black line) remains similar (Figure 5B). For the second specimen, the heating rate was 4 s and the cooling rate was 6 s. The multilayer film became more rigid and its response to temperature was less sensitive due to the hardening of the polymeric layers. The sensitivity of the film is different and smaller than the sensitivity at the beginning of the experiment. The hardening of the material is also related to the heating and cooling rate; therefore, lower rates would lead to smaller shape shifts because of the material degradation. Crystalline polymers develop a partially crystalline structure upon cooling with the cooling rate affecting the rigidity of the material [20,21]. If the cooling rate is very low, the geometrical changes will be negligible. We did not conduct the experiment at the melting temperature; however, we reached high enough temperatures, namely ≈65 °C. By increasing the temperature near 100 °C, one may clearly observe that the geometry of the specimen will be altered significantly due to the different degree of the crystalline structure.

#### 3.1.3. Aluminum Strips (18 μm) on a Trilayer Film with an Aluminum Substrate

For the third specimen, the distance between the location of the final position (Figure 5C) at the beginning of the test and at the end of the test (after 7000 thermal cycles) changed by B_*Ν* = 1_ − B’_*Ν* = 7000_ = 0.3 mm. For this multilayer material, the heating rate was 4 s and the cooling rate was 8 s. Also, we observe that the inclination of the linear fitting curve (from point B or B’ to point A) remains the same at the beginning (orange line) and at the end of the thermal cycling test (black line).

After 7000 thermal cycles, the tested films were still healthy and no significant failure occurred. Despite the very good performance of the multilayer material, we observed that local degradation may occur because of material imperfections and inaccuracies in the manufacturing process.

### 3.2. Surface and Crack Formation Characterization 

#### 3.2.1. Aluminum Strips (5 μm) on a Polyethylene Substrate

**Figure 6:**
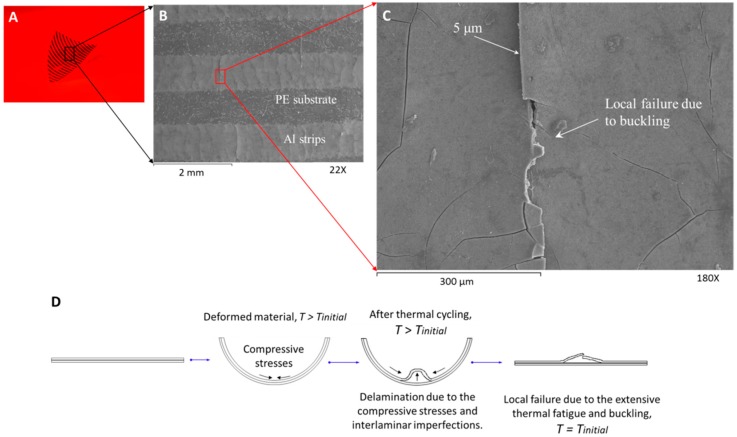
Scanning electron microscopy characterization of the first multilayer film. (**A**) Representative anisotropic film and (**B**,**C**) surface characterization of the first film for less than 100 thermal cycles (aluminum strips of 5 μm thickness), and (**D**) crack formation. The temperature change was ≈35 °C.

Figure 6A shows the transformed shape of the anisotropic film. The thickness of the aluminum strips is approximately 5 μm. We cut smaller specimens (≈11 mm × 11 mm) and we scanned their entire surface using SEM of both the healthy and the fatigued multilayer materials (detail of Figure 3A). Figure 6B,C shows the aluminum strips and the occurred failures at different levels of magnification after few thermal cycles (i.e., *N* < 100). The combination of the small thickness of the strips and their large deformation creates small interlaminar failures. After a few thermal cycles, extensive failures may be observed because of the compressive forces. Consequently, the interlaminar failures of the strips create extensive cracks on the entire material. The crack formation occurs owing to the buckling effects on the thin aluminum strips (Figure 6D).

As the width of the strip decreases, the direction of the crack becomes perpendicular to the direction of the strip. Despite the extensive crack formation, the material remains functional and is capable of achieving large deformations. However, the sensitivity of the film decreased significantly (Figure 5A).

#### 3.2.2. Aluminum Strips (18 μm) on a Polyethylene Substrate

**Figure 7:**
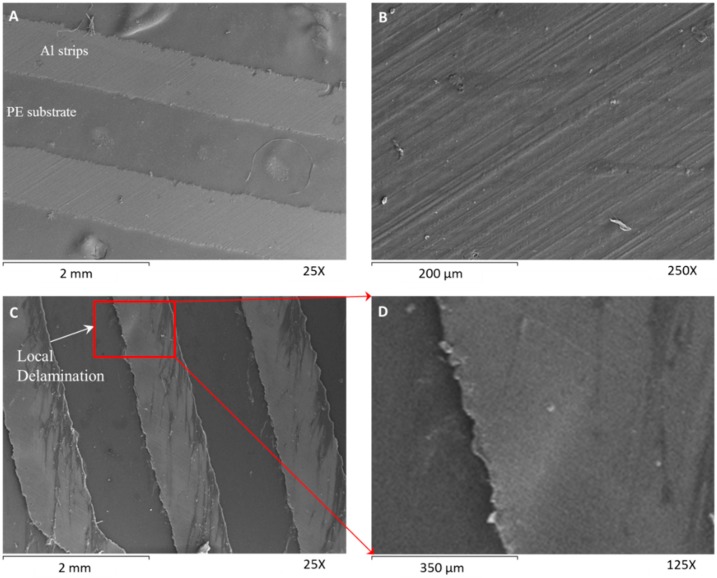
Scanning electron microscopy characterization of the second multilayer film. (**A**,**B**) Untested specimen with zero thermal fatigue cycles. (**A**) Scanning electron microscopy images of the aluminum strips over the PE substrate and (**B**) morphology of the strip’s surface. (**C**,**D**) Tested specimen after 7000 thermal cycles. (**C**) Scanning electron microscopy images of the morphology of the surface aluminum strips over the PE substrate and (**D**) edge delamination of the aluminum strip. The temperature change was ≈45 °C. The two smaller examined specimens were parts of the same multilayer anisotropic film.

Figure 7A shown the formed aluminum strips of the healthy material on the PE layer. The thickness of the aluminum film is 18 μm. We did not observe any delamination or crack on the healthy material (zero thermal cycles) (Figure 7B). Only small defects exist owing to manufacturing inaccuracies. After 7000 thermal cycles, the tested specimen remains functional without significant loss in its performance (Figure 7C). Small delaminations (interlaminar failures) were observed at the edges of the strips (Figure 7C,D) due to the poor adhesion of the strips with the PE. These small delaminated regions have occurred owing to the compressive stresses and small imperfections. However, in the examined specimen with dimensions of ≈11 mm × 11 mm, we did not observe any cracks. Moreover, only five interlaminar failures were observed.

These interlaminar failures can be eliminated if we increase the width of the strips. Moreover, the delaminated regions could be minimized with the addition of an extra aluminum layer between the strips and the oriented PE. In this case, the shear strength of the adhesion between the strips and the substrate would be significantly higher.

#### 3.2.3. Aluminum Strips (18 μm) on a Trilayer Film with an Aluminum Substrate

In addition, a different film was developed in order to characterize its performance and any possible failure. In this case, the aluminum strips have been adhered on an aluminum substrate and not directly on the polyethylene’s surface.

**Figure 8:**
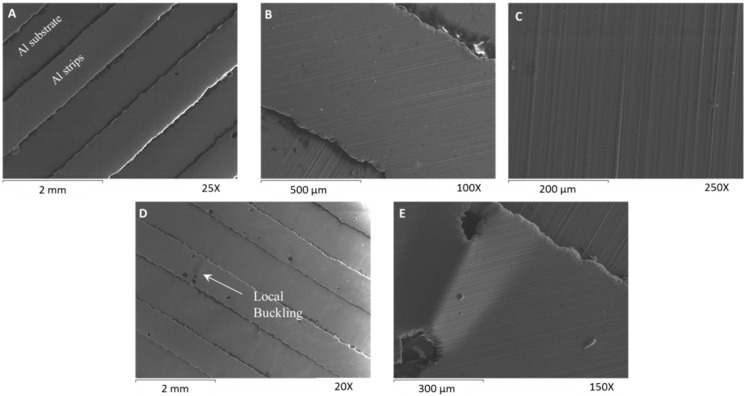
Scanning electron microscopy characterization of the third multilayer film. (**A**–**C**) Untested specimen with zero thermal fatigue cycle. (**A**) Morphology of the aluminum strips’ surface, (**B**) magnification of an aluminum strip, and (**C**) detail of the aluminum strip’s surface. (**D**,**E**) Tested specimen after 7000 thermal cycles. (**D**) Morphology of the aluminum strips’ surface over the aluminum substrate and (**E**) edge delamination of the aluminum strip. The temperature change was ≈45 °C. The two smaller examined specimens were parts of the same multilayer anisotropic film.

Figure 8A shows the formed aluminum strips of the healthy material on the aluminum layer. The thickness of the aluminum film and that of the aluminum strips is 18 μm. On the healthy specimen, we did not observe any delaminations or cracks (zero thermal cycles) (Figure 8A–C). Only, small defects exist due to manufacturing inaccuracies. After 7000 thermal cycles, the material remains functional without significant loss in its performance (Figure 5C). Small delaminations may be observed at the edges of the strips due to the compressive forces. However, in the smaller examined specimen with dimensions of ≈11 mm × 11 mm, we did not observe any cracks. Only two delaminated regions were observed (Figure 8D,E). The material remains fully functional after 7000 thermal cycles and is capable of achieving large deformations while maintaining its exact geometry during the heating or cooling stage (Figure 5C).

Very thin aluminum substrates lead to extensive crack formation, whereas thicker aluminum strips prevent the formation of extensive failures. The most important limitation is related to the type of the polymeric material, since the maximum operational temperature determines the durability and the repeatability of the shape transformation. The design of the higher operational temperature as well as the lower operational temperature of these materials is expected to be more versatile because we can use different polymeric materials (e.g., polyether ether ketone (PEEK) or polytetrafluoroethylene (PTFE)) that may allow us to operate at temperatures above 200 °C, as well as at cryogenic temperatures (<100 K) [22]. The mechanical properties of PEEK and PTFE are not easily compromised, and the materials remain unaffected. Moreover, most engineering plastics are generally well-suited to very low temperatures. The aforementioned thermoplastics could be used for very low to very high temperatures.

## 4. Conclusions

Nonliving tissues drastically alter their geometry using only their multilayer and anisotropic structure. Similarly, complex movements and large deformations in response to a stimulus can be realized using common/commercial multilayer materials. The shape-shifting of the material can be triggered by temperature stimuli. The spatial distribution of the strips on the PE surface determines the manner in which they transform their structure. By controlling the direction and the distribution of the strips, we can design a material that can form a circle, a helix or more complex shapes.

The resistance of these bioinspired films in thermal cycling is strongly related to the thickness of the strip and the type of the substrate. Very thin aluminum strips cannot withstand the excessive thermal fatigue owing to the compression forces. Small imperfections and poor adhesion initiate small delaminations and buckling failures after a few thermal cycles. In contrast, thicker aluminum strips show excellent resistance to thermal fatigue after a few thousand thermal cycles. This fabrication process may become upscaled because (i) the multilayer material consists of common thermoplastic materials, and (ii) the hot and cold rolling pressing techniques, as well as the etching techniques, are used in large industries and may lead to the fabrication of inexpensive responsive materials in the building sector [23,24], sensors, robotics [12], light control [23,24], passive thermal control in space applications [16], and additive manufacturing.

## Supplementary Materials

The following are available online at http://www.mdpi.com/2313-7673/3/3/20/s1. Video S1: Bending movement at the 0° direction (speed ×4), Video S2: Bending movement at the 45° direction (speed ×4), Video S3: Transformation of a flat strip to a helix (speed ×3).
